# Three new fossil records of *Equisetum* (Equisetaceae) from the Neogene of south-western China and northern Vietnam

**DOI:** 10.3897/phytokeys.138.38674

**Published:** 2020-01-10

**Authors:** Aye Thida Aung, Jian Huang, Truong Van Do, Ai Song, Jia Liu, Zhe-Kun Zhou, Tao Su

**Affiliations:** 1 CAS Key Laboratory of Tropical Forest Ecology, Xishuangbanna Tropical Botanical Garden, Chinese Academy of Sciences, Mengla 666303, China Xishuangbanna Tropical Botanical Garden, Chinese Academy of Sciences Mengla China; 2 University of Chinese Academy of Sciences, Beijing 100049, China University of Chinese Academy of Sciences Beijing China; 3 Southeast Asia Biodiversity Research Institute, Chinese Academy of Sciences, Yezin, Nay Pyi Taw 05282, Myanmar Southeast Asia Biodiversity Research Institute, Chinese Academy of Sciences Yezin China; 4 Vietnam National Museum of Nature, Vietnam Academy of Science and Technology, Hanoi, 100803, Vietnam Vietnam National Museum of Nature, Vietnam Academy of Science and Technology Hanoi Vietnam; 5 Graduate University of Science and Technology, Vietnam Academy of Science and Technology, 18 Hoang Quoc Viet, Hanoi, Vietnam Graduate University of Science and Technology Hanoi Vietnam; 6 Research Center for Earth System Science, Yunnan University, Kunming 650500, China Yunnan University Kunming China; 7 CAS Key Laboratory for Plant Diversity and Biogeography of East Asia, Kunming Institute of Botany, Chinese Academy of Sciences, Kunming 650204, China Southeast Asia Biodiversity Research Institute, Chinese Academy of Sciences Yezin Myanmar

**Keywords:** Diversity, *
Equisetum
*, Miocene, Pliocene, rhizome tubers

## Abstract

Three fossil species of *Equisetum* (Equisetaceae) were reported from the Neogene of south-western China and northern Vietnam, based on well-preserved rhizomes with tubers. Equisetum cf. pratense Ehrhart from the middle Miocene of Zhenyuan County, Yunnan Province, China is characterised by a bunch of three ovate tubers with longitudinal ridges on the surface. *Equisetum yenbaiense* A.T. Aung, T. Su, T.V. Do & Z.K. Zhou, **sp. nov.** from the late Miocene of Yenbai Province, Vietnam is characterised by four bunches of elongate tubers arranged in a whorl on a node. *Equisetum yongpingense* A.T. Aung, T. Su & Z.K. Zhou, **sp. nov.** from the late Pliocene of Yunnan is characterised by fibrous roots on most nodes and two to four bunches of large cylindrical tubers arranged in a whorl on a node. Floristic assemblages suggest that these species might have grown near a riverside or lakeshore. These new fossil records improve our understanding of species richness of *Equisetum* and their distribution range during the Neogene in Asia.

## Introduction

The order Equisetales, including Calamitaceae, Tchernoviaceae, Gonduanostachyaceae and Equisetaceae, has a very long evolutionary history that can be dated back to the Devonian (Gu and Shi 1974; Wang et al. 2005). Amongst the families, only Equisetaceae is extant. The earliest fossil record of Equisetaceae could be traced back to the Carboniferous, with *Equisetites hemingwayii* from the early Pennsylvanian of Yorkshire, UK (Kidston 1883). *Equisetum* (Horsetails) is the only living genus in the family Equisetaceae, with about 15 species distributed widely around the world, except for Antarctica (Kenrick and Crane 1997). It is characterised by hollow aerial stems with nodes and reduced leaves, which are similar to their arborescent ancestors. *Equisetum laterale* from the Middle Triassic of Australia is the oldest fossil species in *Equisetum* (Gould 1968). Even some *Equisetum*-like fossils from the Mesozoic have been identified as *Equisetum*, the divergence of *Equisetum* species occurred during the late Eocene (~40 Ma) according to molecular data, with the main radiation during the Neogene (Des Marais et al. 2003; Elgorriaga et al. 2018).

In *Equisetum* fossils, tuberous rhizomes are the most commonly preserved organ in the Cenozoic strata around the world, such as in North America (Lesquereux 1878; Bell 1949; Becker 1969; Skog and Dilcher 1994), Europe (Watson and Batten 1990; Denk et al. 2005) and Asia (Kon’no 1962; Wu 1999; Sun et al. 2001). In China, the fossil records are rich, but most fossils are limited in northern China, for examples, in the Lower Cretaceous Yixian Formation and Fuxin Formation in Liaoning (Chen et al. 1988; Sun et al. 2001), the Lower Cretaceous Muling Formation of Jixi Basin, Heilongjiang (Yang 2003) and the Middle Eocene Hunchun Formation in Jilin (Guo 2000). In south-western China, only four fossil records of *Equisetum* have been found, i.e. Equisetum cf. oppositum from the Paleocene-Eocene of the Niubao Formation in Nima Basin, northern Tibet (Yang et al. 2016), *Equisetum oppositum* from the Lower Oligocene Lawula Formation in eastern Tibet (Ma et al. 2012), Equisetum cf. pratense from the Lower Oligocene of Lühe coal-mine in south-central Yunnan (Zhang et al. 2007) and *Equisetum* sp. from the Middle Miocene Wulong Formation in southern Tibet (Geng and Tao 1982).

Although some species of *Equisetum* still survive in Asia nowadays, such as *Equisetum diffusum*, *Equisetum hyemale* and *Equisetum pratense*, its fossil records remain limited, especially during the Neogene. In this study, we describe three fossil species of *Equisetum* from the Neogene of south-western China and northern Vietnam. Further, we discuss the ecological and biogeographic implications, based on these new fossil records.

## Materials and methods

### Fossil localities

**Figure 1.**
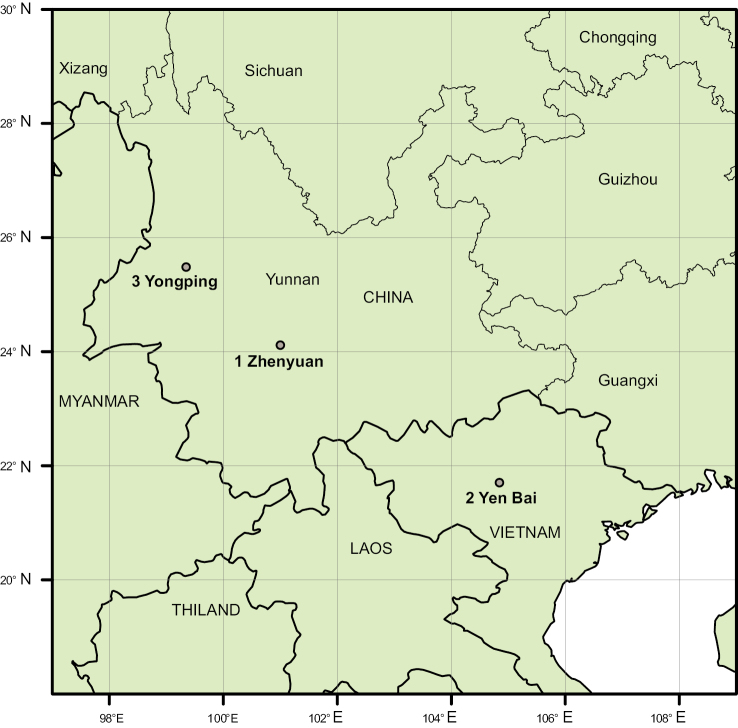
Map showing the locations of the fossils collected in this study. 1. Sanzhangtian, Zhenyuan County, Yunnan, south-western China (the middle Miocene); 2. Hop Thanh Village, Tuy Loc Commune, Yen Bai Province, northern Vietnam (the late Miocene); 3. Longmen, Yongping County, Yunnan Province, south-western China (the late Pliocene).

**Zhenyuan, south-western China (the middle Miocene).** A fossil was found in the Dajie Formation, Sanzhangtian Village, Zhenyuan County, central Yunnan Province, south-western China (Fig. 1). The Dajie Formation, which is mainly distributed in central Yunnan, is assigned to the middle Miocene in age, based on lithological and palynological comparisons (Bureau of Geology and Mineral Resources of Yunnan Province (BGMRYP) 1990). Fossil in this study is from light yellow mudstone of the upper layer in the stratum. Many plant fossil species have been previously reported from the same site, including *Palaeosorum ellipticum* (Jacques et al. 2013), *Bambusium angustifolia*, *B. latifolia*, *Bambusiculmus angustus*, *B. latus* (Wang et al. 2013), *Celastrus caducidentatus* (Liang et al. 2016a), *Populus zhenyuanensis* (Liang et al. 2016b), *Cladium zhenyuanensis* (Liang et al. 2017), *Zygogynum poratus* (Liang et al. 2018), and Metasequoia cf. glyptostroboides (Wang et al. 2019).

**Yen Bai, northern Vietnam (the late Miocene).** Fossils were found in the Co Phuc Formation, Hop Thanh Village, Tuy Loc Commune, Yen Bai Province, northern Vietnam (Fig. 1). The fossil site is situated in the Yen Bai Basin along the Red River Fault Zone, which is a main Cenozoic strike-slip zone in Southeast Asia. The Co Phuc Formation belongs to the late Miocene in age which mainly consists of siltstone and fine sandstone (Wysocka and Świerczewska 2010). The fossils included in this study were collected from the yellow siltstone of the upper layer. Plant fossils such as Polypodiaceae, Palmae and Lauraceae have been previously reported from the fossil site (Zeiller 1903; Colani 1920).

**Yongping, south-western China (the late Pliocene).** The fossils were found in the Sanying Formation, Longmen Village, Yongping County, Yunnan Province, south-western China (Fig. 1). According to a recent paleomagnetic study, the Sanying Formation was dated from the late Miocene to the early Pleistocene (Li et al. 2013). This formation is widely distributed in western and north-western Yunnan Province (BGMRYP 1990). Fossils here are from siltstone in the middle part of the stratum, which is dominated by Quercus section Heterobalanus (evergreen sclerophyllous oak) (Su 2010). Some other species, including *Drynaria callispora* (Su et al. 2011) and *Cedrus angusta* (Su et al. 2013b) have been reported from the same site. According to paleoclimate reconstruction using leaf assemblage of the flora, a warmer and more humid climate than nowadays existed in western Yunnan during the late Pliocene (Su et al. 2013a).

### Morphological observation

*Equisetum* fossils were imaged to view gross morphology by using a digital camera (Nikon D700) with a Kaiser 5510 stand and oblique light. To observe morphological characters in detail, fossils were photographed by stereoscope microscopes (Leica A8APO and ZEISS Smart Zoom 5). The contrast of images was slightly adjusted using the software Adobe Photoshop (version CC 2018). Morphological characters were measured by ImageJ (version 1.52). For comparison with previously published fossil taxa, we checked fossil records from online databases, for examples, Web of Science and Google Scholar. All fossil specimens in this study are deposited in the Paleoecology Collections, Xishuangbanna Tropical Botanical Garden, Chinese Academy of Sciences and Vietnam National Museum of Nature, Vietnam Academy of Science and Technology.

## Results

Order Equisetales Dumortier

Family Equisetaceae A. Michaux ex Alph. De Candolle

Genus *Equisetum* Linnaeus

### *Equisetum* fossils from Zhenyuan, south-western China

#### Equisetum cf. pratense

Taxon classificationPlantaeEquisetalesEquisetaceae

Ehrhart

9F5E1938-99AA-5420-A83C-8736C142C4DD

##### Specimens checked.

**Figure 2.**
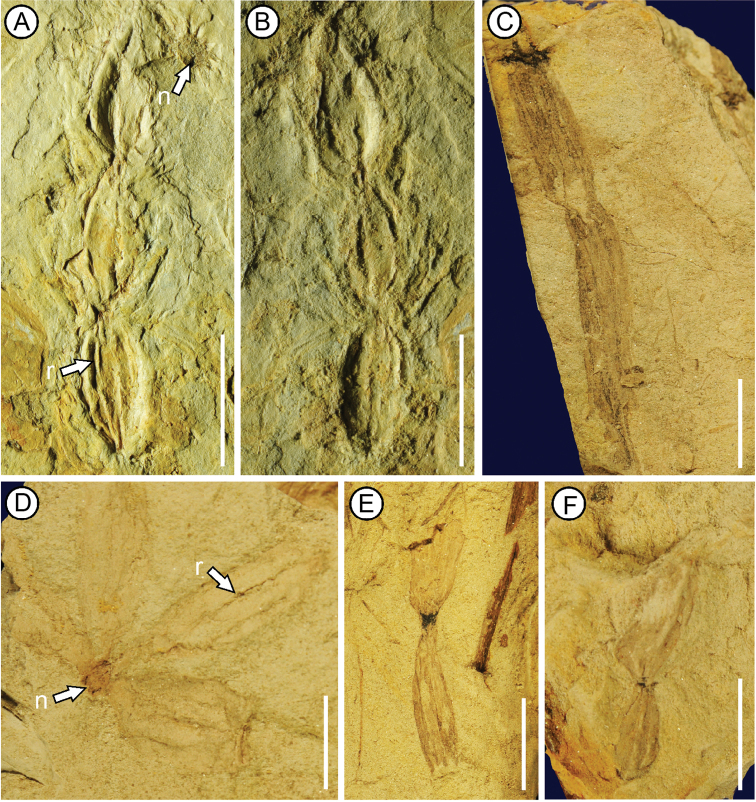
**A–B**Equisetum cf. pratense Ehrhart **C–F***Equisetum yenbaiense* A.T.Aung, T.Su, T.V.Do & Z.K.Zhou, sp. nov. Specimen numbers: **A–B** XTBGSZTF0001 (counterparts) **C** XTBGVNMN4002 **D**&nbsp;XTBGVNMN4001 **E** XTBGVNMN4003 **F** XTBGVNMN4004. **n** = node; **r** = ridge. Scale bars: 1cm.

XTBGSZTF0001 (Fig. 2A–B)

##### Locality.

Dajie Formation, Sanzhangtian Village, Zhenyuan County, central Yunnan Province, South-western China (24.100N, 101.216E).

##### Age.

The middle Miocene.

##### Repository.

Paleoecology Collections, Xishuangbanna Tropical Botanical Garden, Chinese Academy of Sciences.

##### Description.

Only one bunch of tubers are preserved on the specimen, tubers are ovate in shape, three tubers of equal size are arranged in one row (Fig. 2A–B). The length and width of the tubers are ~ 0.9 to 1.2 cm and 0.6 to 0.8 cm, respectively. Two to three longitudinal ridges are present on the surface of each tuber (Fig. 2A–B). The tip of the tuber is mucronate (Fig. 2A–B). These characters fit well within the morphology of E. cf. pratense, a fossil species reported from the early Oligocene in the Lühe coal-mine, south-central Yunnan (Zhang et al. 2007; Linnemann et al. 2018).

### *Equisetum* fossils from Yen Bai, northern Vietnam

#### Equisetum yenbaiense

Taxon classificationPlantaeEquisetalesEquisetaceae

A.T.Aung, T.Su, T.V.Do & Z.K.Zhou sp. nov.

8AF9194F-9E0C-55B4-A5EA-87D79B3ACA15

##### Holotype.

XTBGVNMN4001 (Fig. 2D).

##### Paratypes.

XTBGVNMN4002-4004 (Fig. 2C, E–F).

##### Locality.

Hop Thanh Village, Tuy Loc Commune, Yen Bai Province, northern Vietnam (21.725N, 104.849E).

##### Age.

The late Miocene.

##### Repository.

Paleoecology Collections, Xishuangbanna Tropical Botanical Garden, Chinese Academy of Sciences and Vietnam National Museum of Nature, Vietnam Academy of Science and Technology.

##### Etymology.

The species name ‘*yenbaiense*’ means that fossils are from Yen Bai Province, northern Vietnam.

##### Diagnosis.

Rhizomes with internodes and nodes, node round; Four bunches of tubers arranged in a whorl on a node (Fig. 2D); most tubers elongate in shape, with one to two tubers in each bunch (Fig. 2C–D); longitudinal ridges on the surface of tuber; the tip of tuber mucronate (Fig. 2F).

##### Description.

Rhizomes have both internodes and nodes (Fig. 2D). The internode is ~0.2–0.3 cm in width, the length could not be observed, three longitudinal ridges are on the surface of the internode (Fig. 2D). The node is round and ~0.2 cm in diameter (Fig. 2D). Four bunches of tubers attach to one node and arrange in a whorl (Fig. 2D). Only one tuber is preserved on each bunch, but it is likely that there are more than one tuber on each bunch (Fig. 2E–F). Most tubers are elongate (Fig. 2D); few are elliptical (Fig. 2F), being~ 0.8 to 3.0 cm long and 0.4 to 1.0 cm wide. Two to four ridges are on the surface of each tuber (Fig. 2D). The tip of tuber is mucronate (Fig. 2F).

### *Equisetum* fossils from Yongping, South-western China

#### Equisetum yongpingense

Taxon classificationPlantaeEquisetalesEquisetaceae

A.T.Aung, T.Su & Z.K. Zhou sp. nov.

5610C311-C5DB-5068-AD03-CFF770BF5CA2

##### Holotype.

**Figure 3.**
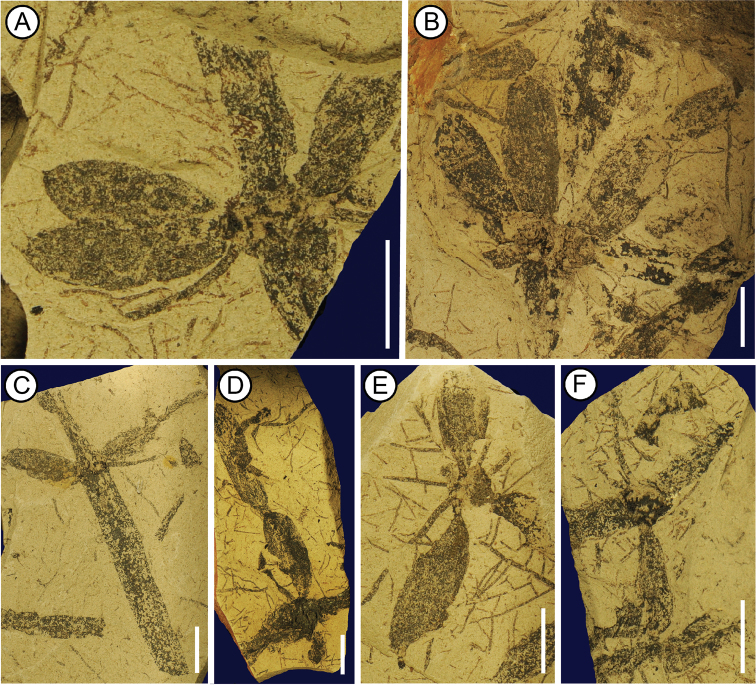
*Equisetum yongpingense* A.T.Aung, T.Su & Z.K.Zhou, sp. nov. Specimen numbers: **A** XTBGYP0748 **B** XTBGYP0747 **C** XTBGYP1014 **D** XTBGYP1015 **E** XTBGYP0750 **F** XTBGYP0749. Scale bars: 1cm.

XTBGYP0748 (Fig. 3A).

##### Paratypes.

XTBGYP0747 (Fig. 3B), XTBGYP1014 (Fig. 3C), XTBGYP1015 (Fig. 3D), XTBGYP0750 (Fig. 3E), XTBGYP0749 (Fig. 3F).

##### Locality.

Sanying Formation, Longmen Village, Yongping County, western Yunnan Province, south-western China (25.518N, 99.519E).

##### Age.

The late Pliocene.

##### Repository.

Paleoecology Collections, Xishuangbanna Tropical Botanical Garden, Chinese Academy of Sciences.

##### Etymology.

The species name ‘*yongpingense*’ means that fossils are from Yongping County, south-western China.

##### Diagnosis.

Rhizomes with internodes and round nodes; fibrous roots on most nodes; two to four bunches of tubers arranged in a whorl on a node (Fig. 3A); tubers cylindrical in shape, one to two tubers on each bunch (Fig. 3A–B); longitudinal ridges not observed on the surface of tuber; the tip of tuber mucronate (Fig. 3A).

##### Description.

Rhizomes have both internodes and nodes (Fig. 3A). The internode is ~0.5 to 0.8 cm wide, the length is up to 5.1 cm (Fig. 3A). The node is round and ~0.7 to 1.0 cm in diameter (Fig. 3A–B). Most nodes have fibrous roots ~ 0.1 to 0.2&nbsp;cm wide. Two to six bunches of tubers attach on one node and are arranged in a whorl (Fig. 3A–B). One to two tubers are preserved on each bunch (Fig. 3A–B). Tubers are cylindrical, ~ 1.5 to 3.4 cm long and 0.6 to 1.2 cm wide (Fig. 3A–F). Ridges were not observed on the surface of each tuber (Fig. 3A–B, E). The tip of tuber is mucronate (Fig. 3A).

## Discussion

**Figure 4.**
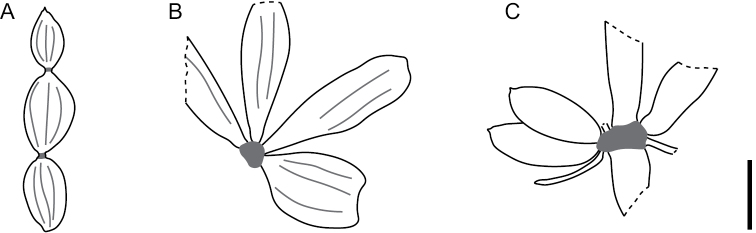
Reconstruction of *Equisetum* from **A** Zhenyuan, south-western China, specimen number XTBGSZTF0001 **B** Yanbei, northern Vietnam, specimen number XTBGVNMN4001; and **C** Yongping, south-western China, specimen number XTBGYP0748. Scale bar: 1.5 cm.

In this study, we reported three new fossil records of *Equisetum* from south-western China and northern Vietnam, based on well-preserved rhizomes with tubers. In *Equisetum*, tubers are the most commonly preserved organ in the fossil records. They are mainly characterised by either a single tuber or one bunch of tubers with longitudinal ridges on the surface, present on fossils we collected (Figs 2–4). These three new fossil records vary in morphological characters, such as the shape and size of tubers (Table 1). They are also different from living species of *Equisetum*, for example, *Equisetum pratense*, as they have lager tuber sizes, based on available information (Zhang et al. 2007; Sun et al. 2013).

**Table 1.** Morphological comparisons between new fossil records in this study and previously reported *Equisetum* and *Equisetites* fossil species.

Species	Tuber arrangement	Number of tubers per bunch	Shape of tuber	Tuber length (cm)	Tuber width (cm)	Age	Locality	References
*Equisetum* sp.	?	3	Elliptical	1.0–1.2	0.6–0.8	Miocene	Iceland	Denk et al. 2005
Equisetum cf. arcticum	Whorl	2–3	Elongate, ovate, round	1.5–2.5	0.5–0.7	Oligocene to Miocene	America	Becker 1969
*Equisetum hunchunense*	Whorl	2–3	Elliptical, ovate	1.5	0.4–0.9	Eocene	China	Guo 2000
*Equisetum jiuquanense*	Single to acervate	2–3	Elliptical, ovate, round	0.3–0.8	0.4–0.7	Early Cretaceous	China	Sun et al. 2013
*Equisetites longevaginatus*	Single or opposite	2–3	Elliptical, round	0.5–0.8	0.3–0.5	Cretaceous	China	Sun et al. 2001
*Equisetum oppositum*	Opposite	6	Round, elliptical, ovate	1.0–1.6	0.8–1.2	Early Oligocene	China	Ma et al. 2012
Equisetum cf. oppositum	Single	5–6	Elliptical, ovate, and nearly round	1.0–1.6	0.8–1.2	Paleocene-Eocene	China	Yang et al. 2016
Equisetum cf. pratense	?	3	Ovate	0.9–1.2	0.6–0.8	Middle Miocene	China	This study
*Equisetites vittatus*	Whorl	1–2	Elliptical, elongate	0.8–3.0	0.4–1.0	Late Miocene	Vietnam	This study
*Equisetum yongpingense*	Whorl	1–2	Cylindrical	1.5–3.4	0.6–1.2	Late Pliocene	China	This study

Equisetum cf. pratense from the middle Miocene of Zhenyuan, south-western China have an ovate tuber shape, which is similar to most fossil records of *Equisetum*, but different from some fossil species, for example, the tubers of *Equisetites longevaginatus* are elliptical or round in shape (Table 1). The size of tubers in E. cf. pratense is smaller than other fossil species, such as *Equisetum oppositum*, Equisetum cf. oppositum and *Equisetum hunchunense* (Table 1). Generally, all observed morphological characters of the fossil, such as the size and shape of tubers, as well as the mucronate tip of tubers, were in accordance with E. cf. pratense, a fossil species previously reported from the early Oligocene of the Lühe coal-mine, south-central Yunnan (Zhang et al. 2007). However, we only found one bunch of tubers at the fossil site, whereas the fossils from the Lühe coal-mine have one to four bunches of tubers with an acervate arrangement on rhizome nodes. More fossils are needed to further determine the arrangement of tubers to better understand the systematic relationship between these two fossil records. Judging by the high morphological similarity, we assign the fossil from Zhenyuan as E. cf. pratense.

For *Equisetum yenbaiense* from the late Miocene of northern Vietnam, the tuber arrangement is a whorl on a rhizome node, which also occurs in *E. hunchunense* and E. cf. arcticum. However, both *E. hunchunense* and E. cf. arcticum have two to three tubers per bunch, whereas *E. yenbaiense* only has one or two tubers per bunch, which might be partly due to the preservation of the fossil. In addition, there are more than three tubers per bunch in *E. oppositum* and E. cf. oppositum (Table 1). The shape of tubers in *E. yenbaiense* is elongate, the ratio between length and width is higher than most other fossil species, except for E. cf. arcticum. We noted that the tuber size in *E. yenbaiense* is larger than that in E. cf. arcticum (Table 1). Therefore, we designate the new fossil specimens from northern Vietnam as a new species, namely, *E. yenbaiense* A.T. Aung, T. Su, T.V. Do & Z.K. Zhou, sp. nov.

In *Equisetum yongpingense*, there are two to four bunches of tubers arranged in a whorl on a node, which has the same pattern as *E. hunchunense* and E. cf. arcticum. The tip of the tubers is mucronate in *E. yongpingense*, which is also present on other previously reported fossil species (Table 1). However, the tuber shape of *E. yongpingense* is cylindrical, which is different from other fossil records. Ridges on the surface of tubers are not prominent in *E. yongpingense*, whereas ridges are present in other fossil species. In *E. yongpingense*, fibrous roots were also observed on the rhizome node, which is not observed on other fossil species. Therefore, we name these fossil specimens from Yongping as *E. yongpingense* A.T.Aung, T.Su & Z.K.Zhou, sp. nov.

The three new fossil records described in this study expand the distribution of *Equisetum* during the Neogene in Asia. These findings and previous fossil records indicate that *Equisetum* has been widely distributed in Asia since the Paleocene and become diverse in Asia since the Oligocene. According to fossil assemblages, *Equisetum* mainly grew under wet conditions. Other taxa that were reported from the same stratum in Zhenyuan, such as *Palaeosorum ellipticum* (Jacques et al. 2013), *Bambusium angustifolia*, *B. latifolia*, *Bambusiculmus angustus*, *B. latus* (Wang et al. 2013) and Metasequoia cf. glyptostroboides (Wang et al. 2019), tend to grow near the riverside or lakeshore. Therefore, we considered that E. cf. pratense might favour a similar habitat. In addition, *E. yongpingense* was found in the Upper Pliocene of western Yunnan, where numerous fruits from *Trapa*, an aquatic plant, had also been found in the same layer (Su 2010). Therefore, this habit might have persisted throughout the Cenozoic. Tubers indicate a perennial living form and should contribute to the adaptation of *Equisetum* under seasonally unfavourable conditions by storing starch (Taiz and Zeiger 2010). Eventually, tubers became important elements in local vegetation, which was evidenced by the abundance of specimens in many fossil sites (Becker 1969; Guo 2000; Ma et al. 2012; Yang et al. 2016).

## Supplementary Material

XML Treatment for Equisetum cf. pratense

XML Treatment for Equisetum yenbaiense

XML Treatment for Equisetum yongpingense
